# Crystal structures of 4-meth­oxy­benzoic acid–1,3-bis­(pyridin-4-yl)propane (2/1) and biphenyl-4,4′-di­carb­oxy­lic acid–4-meth­oxy­pyridine (1/2)

**DOI:** 10.1107/S2056989017010167

**Published:** 2017-07-17

**Authors:** Kazuma Gotoh, Hiroyuki Ishida

**Affiliations:** a Department of Chemistry, Faculty of Science, Okayama University, Okayama 700-8530, Japan

**Keywords:** crystal structure, 4-meth­oxy­benzoic acid, 1,3-bis­(pyridin-4-yl)propane, biphenyl-4,4′-di­carb­oxy­lic acid, 4-meth­oxy­pyridine

## Abstract

The crystal structures of two hydrogen-bonded compounds, namely 4-meth­oxy­benzoic acid–1,3-bis­(pyridin-4-yl)propane (2/1), C_13_H_14.59_N_2_·C_8_H_7.67_O_3_·C_8_H_7.74_O_3_, (I), and biphenyl-4,4′-di­carb­oxy­lic acid–4-meth­oxy­pyridine (1/2), C_14_H_9.43_O_4_·C_6_H_7.32_NO·C_6_H_7.25_NO, (II), have been determined at 93 K. In (I), the asymmetric unit consists of two crystallographically independent 4-meth­oxy­benzoic acid mol­ecules and one 1,3-bis­(pyridin-4-yl)propane mol­ecule. The asymmetric unit of (II) comprises one biphenyl-4,4′-di­carb­oxy­lic acid mol­ecule and two independent 4-meth­oxy­pyridine mol­ecules. In each crystal, the acid and base mol­ecules are linked by short O—H⋯N/N—H⋯O hydrogen bonds, in which H atoms are disordered over the acid O-atom and base N-atom sites, forming a linear hydrogen-bonded 2:1 or 1:2 unit of the acid and the base. The 2:1 units of (I) are linked *via* C—H⋯π, π–π and C—H⋯O inter­actions into a tape structure along [101], while the 1:2 units of (II) form a double-chain structure along [-101] through π–π and C—H⋯O inter­actions.

## Abstract 2

Crystal structures of the title hydrogen-bonded compounds have been determined at 98 K. In each crystal, the acid and base mol­ecules are linked by short O—H⋯N/N—H⋯O hydrogen bonds, forming a linear hydrogen-bonded 2:1 or 1:2 unit of the acid and the base.

## Chemical context   

Co-crystals of the 4-alk­oxy­benzoic acid–4,4′-bipyridyl (2/1) and 4-alk­oxy­benzoic acid–(*E*)-1,2-bis­(pyridin-4-yl)ethene (2/1) systems show thermotropic liquid crystallinity (Kato *et al.*, 1990 ▸, 1993 ▸; Grunert *et al.*, 1997 ▸). Of these co-crystals, the crystal structures of 4,4′-bipyridyl with 4-meth­oxy­benzoic acid (Mukherjee & Desiraju, 2014 ▸; Ramon *et al.*, 2014 ▸), 4-eth­oxy, 4-(*n*-prop­oxy)- and 4-(*n*-but­oxy)benzoic acid (Tabuchi *et al.*, 2015*a* ▸), and the crystal structures of (*E*)-1,2-bis­(pyridin-4-yl)ethene with 4-meth­oxy-, 4-eth­oxy-, 4-(*n*-prop­oxy)-, 4-(*n*-but­oxy)-, 4-(*n*-pent­yloxy)- and 4-(*n*-hex­yloxy)benzoic acid (Tabuchi *et al.*, 2016*a* ▸,*b* ▸) have been reported. In these crystals, the two acids and the base are held together by short inter­molecular O—H⋯N hydrogen bonds, forming linear 2:1 units of the acid and the base. As an expansion of our work on the structural characterization of hydrogen-bonded co-crystals with organic acid and base mol­ecules, we have prepared 4-meth­oxy­benzoic acid–1,3-bis­(pyridin-4-yl)propane (2/1), (I), and biphenyl-4,4′-di­carb­oxy­lic acid–4-meth­oxy­pyridine (1/2), (II), and analyzed the crystal structures.

## Structural commentary   

**Figure 1:**
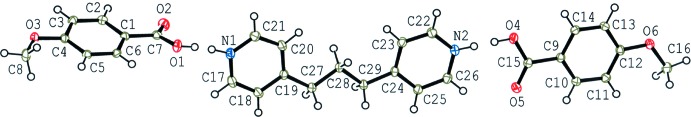
The mol­ecular structure of compound (I), showing the atom-numbering scheme. Displacement ellipsoids of non-H atoms are drawn at the 50% probability level and H atoms are drawn as circles of arbitrary size.

The mol­ecular structures of (I) and (II) are shown in Figs. 1 ▸ and 2 ▸, respectively. The asymmetric unit of (I) consists of two crystallographically independent 4-meth­oxy­benzoic acid mol­ecules and one 1,3-bis­(pyridin-4-yl)propane mol­ecule. The acid and base mol­ecules are held together *via* short O—H⋯N/N—H⋯O hydrogen bonds between the carboxyl O and pyridine N atoms (Table 1 ▸), forming a 2:1 unit. In the hydrogen bonds, the H atoms are disordered over O- and N-atom sites, with occupancy ratios of 0.67 (3):0.33 (3) between atoms O1 and N1, and 0.74 (3):0.26 (3) between atoms O4 and N2. The O1/C7/O2 and N1/C17–C21 planes in one hydrogen-bonded unit are approximately perpendicular to each other, with a dihedral angle of 85.97 (13)°. On the other hand, the O4/C15/O5 and N2/C22–C26 planes in the other hydrogen-bonded unit are approximately planar, with a dihedral angle of 10.18 (14)°, and a weak C—H⋯O hydrogen bond (C26—H26⋯O5) is observed in the hydrogen-bonded unit. The dihedral angles between the pyridine N1/C17–C21 and N2/C22–C26 rings, between the benzene C1–C6 and pyridine N1/C17–C21 planes, and between the benzene C9–C14 and pyridine N2/C22–C26 planes are 8.68 (6), 72.93 (6) and 9.05 (6)°, respectively.
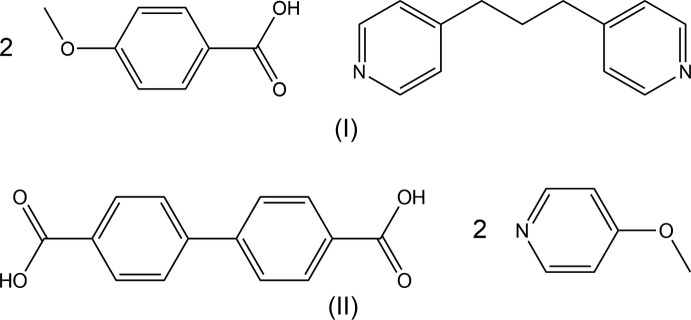


The asymmetric unit of (II) is composed of one biphenyl-4,4′-di­carb­oxy­lic acid mol­ecule and two crystallographically independent 4-meth­oxy­pyridine mol­ecules, and the acid and the two bases are held together by short O—H⋯N/N—H⋯O hydrogen bonds (Table 2 ▸), forming a linear 1:2 aggregate with pseudo-inversion symmetry. Similar to compound (I), the H atoms in the hydrogen bonds are disordered over two sites, with occupancy ratios of 0.68 (3):0.32 (3) between atoms O1 and N1, and 0.75 (3):0.25 (3) between atoms O3 and N2. The hydrogen-bonded 1:2 unit is approximately planar and weak C—H⋯O hydrogen bonds (C19—H19⋯O2 and C25—H25⋯O4) are observed. The dihedral angle between the benzene rings of the biphenyl-4,4′-di­carb­oxy­lic acid is 3.87 (5)°. The N1/C15–C19 pyridine ring makes dihedral angles of 5.62 (12) and 2.55 (5)°, respectively, with the O1/C7/O2 and C1–C6 planes. The N2/C21–C25 pyridine ring makes dihedral angles of 6.84 (12) and 9.50 (5)°, respectively, with the O3/C14/O4 and C8–C13 planes.

## Supra­molecular features   

**Figure 3:**
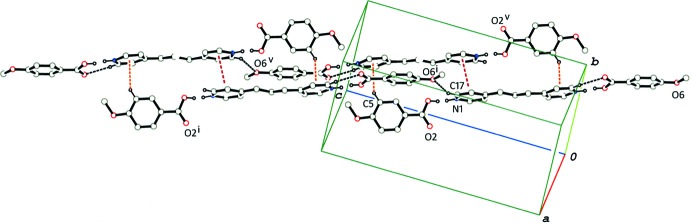
A partial packing diagram of compound (I), showing inversion dimers formed by C—H⋯π inter­actions (orange–red dashed lines) and π–π stacking inter­actions (brown dashed lines), and a tape structure formed by C—H⋯O hydrogen bonds (black dashed lines) between the dimers. H atoms not involved in the above inter­actions and O—H⋯N/N—H⋯O hydrogen bonds have been omitted. [Symmetry codes: (i) *x* + 1, *y*, *z* + 1; (v) −*x*, −*y* + 1, −*z* + 1.]

**Figure 4:**
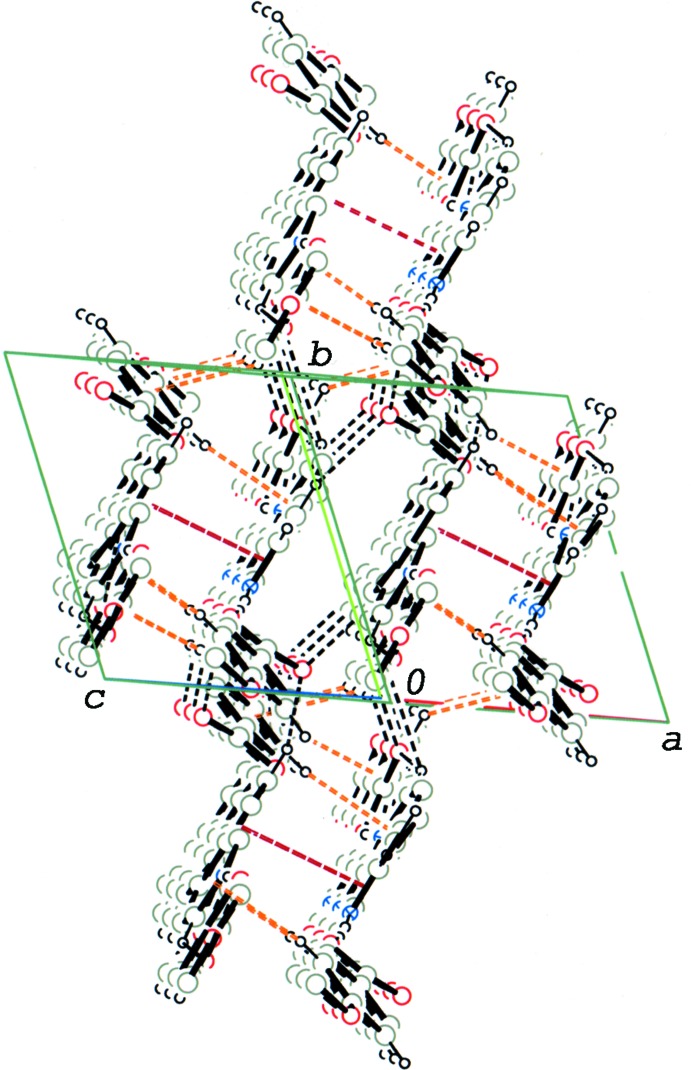
A packing diagram of compound (I), viewed approximately along [101], showing C—H⋯O hydrogen bonds (black dashed lines). C—H⋯π inter­actions (orange–red dashed lines) and π–π stacking inter­actions (brown dashed lines) formed between mol­ecular tapes. H atoms not involved in the above inter­actions have been omitted.

In the crystal of (I), the 2:1 units are linked by a pair of C—H⋯π inter­actions (C5—H5⋯*Cg*2^v^; *Cg*2 in the centroid of the pyridine N2/C22–C26 ring; symmetry code as given in Table 1 ▸), and a π–π inter­action [*Cg*1⋯*Cg*1^v^ = 3.6588 (16) Å; *Cg*1 is the centroid of the pyridine N1/C17–C21 ring], forming an inversion dimer. The dimers are linked *via* C—H⋯O inter­actions (C17—H17⋯O6^i^; Table 1 ▸) into a tape structure running along [101] (Fig. 3 ▸). The tapes running along the same direction are further linked *via* the rest of the C—H⋯O and C—H⋯π inter­actions (Table 1 ▸), forming a three-dimensional network (Fig. 4 ▸).

**Figure 5:**
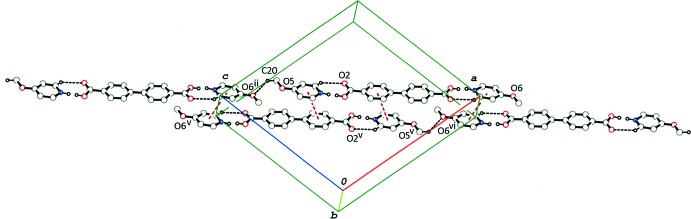
A partial packing diagram of compound (II), showing a double-chain structure formed by O—H⋯N/N—H⋯O hydrogen bonds, C—H⋯O inter­actions (black dashed lines) and π–π stacking inter­actions (brown dashed lines). H atoms not involved in the above inter­actions have been omitted. [Symmetry codes: (ii) *x* − 1, *y*, *z* + 1; (v) −*x* + 1, −*y* + 1, −*z* + 1: (vi) −*x* + 2, −*y* + 1, −*z*.]

**Figure 6:**
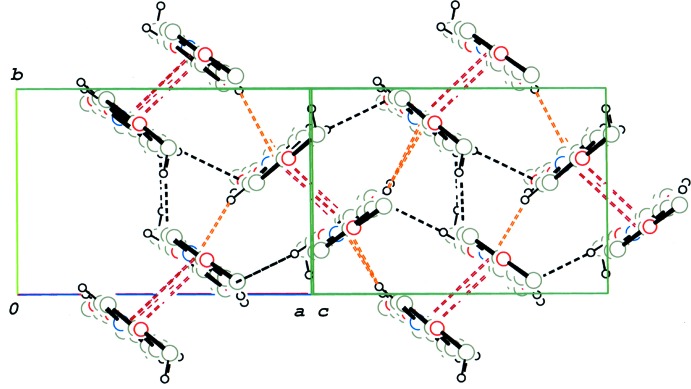
A partial packing diagram of compound (II), viewed along [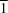
01], showing the arrangement of the mol­ecular chains. C—H⋯O hydrogen bonds, C—H⋯π inter­actions and π–π stacking inter­actions are shown by black, orange–red and brown dashed lines, respectively. H atoms not involved in the above inter­actions have been omitted.

In the crystal of (II), the 1:2 units are linked by a C—H⋯O inter­action (C20—H20*A*⋯O6^ii^; symmetry code as given in Table 2 ▸) into a chain structure along [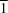
01]. Ajacent chains, which are related by an inversion centre, are further linked *via* π–π inter­actions between pyridine N2/C21–C25 rings [centroid-centroid distance = 3.8113 (13) Å] and between the benzene C1–C6 and pyridine N1/C15–C19 rings [centroid-centroid distance = 3.6613 (12) Å], forming a double-chain structure (Fig. 5 ▸). Weak C—H⋯O and C—H⋯π inter­actions are observed between the double chains (Table 2 ▸) and the 1:2 units are arranged in the crystals with their long axes parallel to each other (Fig. 6 ▸).

## Database survey   

The crystal structures of co-crystals similar to compound (I), namely 4-meth­oxy­benzoic acid–1,2-bis­(pyridin-4-yl)ethane (2/1) (Mukherjee & Desiraju, 2014 ▸), 4-eth­oxy­benzoic acid–1,2-bis­(pyridin-4-yl)ethane (2/1), 4-(*n*-prop­oxy)benzoic acid–bis­(pyridin-4-yl)ethane (2/1) and 4-(*n*-but­oxy)benzoic acid–1,2-bis­(pyridin-4-yl)ethane (2/1) (Tabuchi *et al.*, 2015*b* ▸) have been reported. These compounds also show thermotropic liquid crystallinity (Tabuchi *et al.*, 2015*b* ▸). A search of the Cambridge Structural Database (CSD, Version 5.38, last update February 2017; Groom *et al.*, 2016 ▸) for organic co-crystals or salts similar to compound (II), namely 4,4′-bi­phenyldi­carb­oxy­lic acid with pyridine derivatives, gave three structures, with CSD refcodes ATOJEZ (Gong *et al.*, 2011 ▸), BIKFUX (Cruz *et al.*, 2004 ▸) and MAZYUI (Du *et al.*, 2006 ▸).

## Synthesis and crystallization   

Single crystals of compound (I) were obtained by slow evaporation from an ethanol solution (200 ml) of 1,3-bis­(pyridin-4-yl)propane (100 mg) with 4-meth­oxy­benzoic acid (155 mg) at room temperature. Crystals of compound (I) were obtained by slow evaporation from a 4-meth­oxy­pyridine solution (5 ml) of bi­phenyl-4,4′-di­carb­oxy­lic acid (100 mg) at room temperature.

## Refinement   

Crystal data, data collection and structure refinement details are summarized in Table 3 ▸. All H atoms in compounds (I) and (II) were found in difference Fourier maps. The H atoms in both compounds which are involved in the O—H⋯N/N—H⋯O hydrogen bonds were found to be disordered over two positions in difference Fourier maps. The positional parameters and the occupancy factors were refined with bond-length restraints of O—H = 0.84 (2) Å and N—H = 0.88 (2) Å, and with *U*_iso_(H) = 1.5*U*_eq_(O,N). Other H atoms were positioned geometrically (C—H = 0.95–0.99 Å) and were treated as riding, with *U*_iso_(H) = 1.2*U*_eq_(C) or 1.5*U*_eq_(methyl C). For compound (I), six reflections were omitted in the final refinement owing to poor agreement between the measured and calculated intensities.

## Supplementary Material

Crystal structure: contains datablock(s) I, II, General. DOI: 10.1107/S2056989017010167/lh5847sup1.cif

Structure factors: contains datablock(s) I. DOI: 10.1107/S2056989017010167/lh5847Isup2.hkl

Structure factors: contains datablock(s) II. DOI: 10.1107/S2056989017010167/lh5847IIsup3.hkl

CCDC references: 1560979, 1560978

Additional supporting information:  crystallographic information; 3D view; checkCIF report

## Figures and Tables

**Figure 2:**
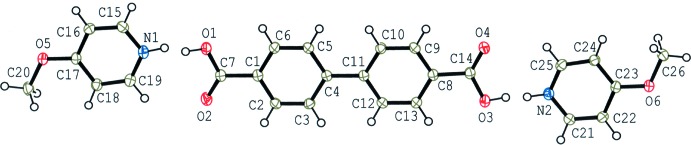
The mol­ecular structure of compound (II), showing the atom-numbering scheme. Displacement ellipsoids of non-H atoms are drawn at the 50% probability level and H atoms are drawn as circles of arbitrary size.

**Table 1:** Hydrogen-bond geometry (Å, °) for (I) *Cg*2, *Cg*3 and *Cg*4 are the centroids of the N2/C22–C26, C1–C6 and C9–C14 rings, respectively.

*D*—H⋯*A*	*D*—H	H⋯*A*	*D*⋯*A*	*D*—H⋯*A*
O1—H1*A*⋯N1	0.87 (2)	1.71 (2)	2.5730 (16)	170 (3)
O4—H4*A*⋯N2	0.88 (2)	1.80 (2)	2.6721 (17)	171 (2)
N1—H1*B*⋯O1	0.89 (2)	1.69 (4)	2.5730 (16)	171 (5)
N2—H4*B*⋯O4	0.87 (2)	1.80 (4)	2.6720 (16)	177 (7)
C17—H17⋯O6^i^	0.95	2.48	3.342 (2)	151
C18—H18⋯O2^ii^	0.95	2.60	3.515 (2)	162
C21—H21⋯O2^iii^	0.95	2.43	3.2563 (19)	145
C26—H26⋯O5	0.95	2.36	3.0890 (18)	133
C3—H3⋯*Cg*4^iv^	0.95	2.64	3.4265 (19)	140
C5—H5⋯*Cg*2^v^	0.95	2.71	3.5440 (18)	146
C10—H10⋯*Cg*3^vi^	0.95	2.89	3.5859 (19)	131
C28—H28*B*⋯*Cg*4^vii^	0.99	2.91	3.7154 (19)	139

Symmetry codes: (i) 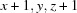; (ii) 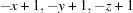; (iii) 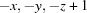; (iv) 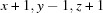; (v) 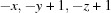; (vi) 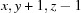; (vii) 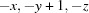.

**Table 2:** Hydrogen-bond geometry (Å, °) for (II) *Cg*2 and *Cg*4 are the centroids of the C8–C13 and N2/C21–C25 rings, respectively.

*D*—H⋯*A*	*D*—H	H⋯*A*	*D*⋯*A*	*D*—H⋯*A*
O1—H1*A*⋯N1	0.87 (2)	1.73 (2)	2.5882 (15)	173 (2)
O3—H3*A*⋯N2	0.87 (2)	1.74 (2)	2.6078 (15)	175 (2)
N1—H1*B*⋯O1	0.88 (2)	1.73 (5)	2.5882 (16)	167 (5)
N2—H3*B*⋯O3	0.88 (2)	1.74 (6)	2.6077 (15)	169 (5)
C10—H10⋯O2^i^	0.95	2.57	3.4146 (17)	148
C19—H19⋯O2	0.95	2.52	3.1901 (17)	128
C20—H20*A*⋯O6^ii^	0.98	2.60	3.3210 (18)	131
C25—H25⋯O4	0.95	2.54	3.2035 (17)	127
C26—H26*B*⋯O4^iii^	0.98	2.43	3.3874 (17)	167
C12—H12⋯*Cg*4^iv^	0.95	2.90	3.6968 (16)	142
C21—H21⋯*Cg*2^iv^	0.95	2.64	3.5284 (16)	155

Symmetry codes: (i) 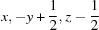; (ii) 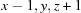; (iii) 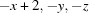; (iv) 
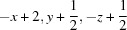
.

**Table 3:** Experimental details

	(I)	(II)
Crystal data		
Chemical formula	C_13_H_14.59_N_2_·C_8_H_7.67_O_3_·C_8_H_7.74_O_3_	C_14_H_9.43_O_4_·C_6_H_7.32_NO·C_6_H_7.25_NO
*M*_r_	502.55	460.47
Crystal system, space group	Triclinic, *P* 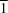	Monoclinic, *P*2_1_/*c*
Temperature (K)	93	93
*a*, *b*, *c* (Å)	7.759 (3), 8.733 (4), 19.904 (7)	18.354 (6), 7.4166 (16), 16.674 (5)
α, β, γ (°)	91.087 (16), 90.593 (17), 113.241 (15)	90, 104.943 (12), 90
*V* (Å^3^)	1238.7 (8)	2192.9 (10)
*Z*	2	4
Radiation type	Mo *K*α	Mo *K*α
μ (mm^−1^)	0.10	0.10
Crystal size (mm)	0.54 × 0.50 × 0.19	0.45 × 0.40 × 0.35

Data collection		
Diffractometer	Rigaku R-AXIS RAPID II	Rigaku R-AXIS RAPID II
Absorption correction	Numerical (*NUMABS*; Higashi, 1999 ▸)	Numerical (*NUMABS*; Higashi, 1999 ▸)
*T*_min_, *T*_max_	0.970, 0.982	0.963, 0.966
No. of measured, independent and observed [*I* > 2σ(*I*)] reflections	12378, 5653, 4561	20851, 5026, 4085
*R*_int_	0.033	0.035
(sin θ/λ)_max_ (Å^−1^)	0.649	0.649

Refinement		
*R*[*F* ^2^ > 2σ(*F* ^2^)], *wR*(*F* ^2^), *S*	0.040, 0.106, 1.07	0.038, 0.108, 1.06
No. of reflections	5653	5026
No. of parameters	350	323
No. of restraints	4	4
H-atom treatment	H atoms treated by a mixture of independent and constrained refinement	H atoms treated by a mixture of independent and constrained refinement
Δρ_max_, Δρ_min_ (e Å^−3^)	0.27, −0.30	0.35, −0.26

Computer programs: *RAPID-AUTO* (Rigaku, 2006 ▸), *Il Milione* (Burla *et al.*, 2007 ▸), *SHELXL2014* (Sheldrick, 2015 ▸), *ORTEP-3 for Windows* (Farrugia, 2012 ▸), *CrystalStructure* (Rigaku, 2010 ▸) and *PLATON* (Spek, 2009 ▸).

## supplementary crystallographic information

### 4-Methoxybenzoic acid–1,3-bis(pyridin-4-yl)propane (2/1) (I) Crystal data

**Table:**

C_13_H_14.59_N_2_·C_8_H_7.67_O_3_·C_8_H_7.74_O_3_	*Z* = 2
*M**_r_* = 502.55	*F*(000) = 532.00
Triclinic, *P*1	*D*_x_ = 1.347 Mg m^−^^3^
*a* = 7.759 (3) Å	Mo *K*α radiation, λ = 0.71075 Å
*b* = 8.733 (4) Å	Cell parameters from 13611 reflections
*c* = 19.904 (7) Å	θ = 3.0–30.1°
α = 91.087 (16)°	µ = 0.10 mm^−^^1^
β = 90.593 (17)°	*T* = 93 K
γ = 113.241 (15)°	Platelet, colorless
*V* = 1238.7 (8) Å^3^	0.54 × 0.50 × 0.19 mm

### 4-Methoxybenzoic acid–1,3-bis(pyridin-4-yl)propane (2/1) (I) Data collection

**Table:**

Rigaku R-AXIS RAPID II diffractometer	4561 reflections with *I* > 2σ(*I*)
Detector resolution: 10.000 pixels mm^-1^	*R*_int_ = 0.033
ω scans	θ_max_ = 27.5°, θ_min_ = 3.0°
Absorption correction: numerical (NUMABS; Higashi, 1999)	*h* = −10→10
*T*_min_ = 0.970, *T*_max_ = 0.982	*k* = −11→11
12378 measured reflections	*l* = −25→25
5653 independent reflections	

### 4-Methoxybenzoic acid–1,3-bis(pyridin-4-yl)propane (2/1) (I) Refinement

**Table:**

Refinement on *F*^2^	Primary atom site location: structure-invariant direct methods
Least-squares matrix: full	Secondary atom site location: difference Fourier map
*R*[*F*^2^ > 2σ(*F*^2^)] = 0.040	Hydrogen site location: mixed
*wR*(*F*^2^) = 0.106	H atoms treated by a mixture of independent and constrained refinement
*S* = 1.07	*w* = 1/[σ^2^(*F*_o_^2^) + (0.0644*P*)^2^] where *P* = (*F*_o_^2^ + 2*F*_c_^2^)/3
5653 reflections	(Δ/σ)_max_ = 0.001
350 parameters	Δρ_max_ = 0.27 e Å^−^^3^
4 restraints	Δρ_min_ = −0.30 e Å^−^^3^

### 4-Methoxybenzoic acid–1,3-bis(pyridin-4-yl)propane (2/1) (I) Special details

**Table:**

Geometry. All esds (except the esd in the dihedral angle between two l.s. planes) are estimated using the full covariance matrix. The cell esds are taken into account individually in the estimation of esds in distances, angles and torsion angles; correlations between esds in cell parameters are only used when they are defined by crystal symmetry. An approximate (isotropic) treatment of cell esds is used for estimating esds involving l.s. planes.
Refinement. Reflections were merged by SHELXL according to the crystal class for the calculation of statistics and refinement. _reflns_Friedel_fraction is defined as the number of unique Friedel pairs measured divided by the number that would be possible theoretically, ignoring centric projections and systematic absences.

### 4-Methoxybenzoic acid–1,3-bis(pyridin-4-yl)propane (2/1) (I) Fractional atomic coordinates and isotropic or equivalent isotropic displacement parameters (Å^2^)

**Table:**

	*x*	*y*	*z*	*U*_iso_*/*U*_eq_	Occ. (<1)
O1	0.13329 (12)	0.23348 (10)	0.63470 (4)	0.02284 (19)	
H1A	0.132 (4)	0.258 (3)	0.5927 (8)	0.034*	0.67 (3)
O2	0.29365 (12)	0.08921 (10)	0.59389 (4)	0.0251 (2)	
O3	0.39277 (11)	0.01871 (10)	0.90676 (4)	0.02030 (18)	
O4	−0.32465 (11)	0.58373 (10)	−0.08794 (4)	0.02144 (18)	
H4A	−0.280 (3)	0.580 (3)	−0.0475 (8)	0.032*	0.74 (3)
O5	−0.10967 (13)	0.84664 (10)	−0.08750 (4)	0.0271 (2)	
O6	−0.44746 (12)	0.77002 (10)	−0.38406 (4)	0.02324 (19)	
N1	0.12203 (14)	0.33437 (12)	0.51474 (5)	0.0211 (2)	
H1B	0.126 (7)	0.290 (5)	0.5542 (14)	0.032*	0.33 (3)
N2	−0.15522 (13)	0.57544 (12)	0.02868 (5)	0.0190 (2)	
H4B	−0.208 (7)	0.577 (7)	−0.0101 (16)	0.028*	0.26 (3)
C1	0.27123 (14)	0.11283 (13)	0.71215 (5)	0.0159 (2)	
C2	0.34417 (15)	−0.00596 (13)	0.72592 (5)	0.0181 (2)	
H2	0.3669 (19)	−0.0701 (16)	0.6893 (7)	0.020 (3)*	
C3	0.38150 (15)	−0.03646 (13)	0.79103 (6)	0.0185 (2)	
H3	0.429441	−0.118989	0.799698	0.022*	
C4	0.34844 (14)	0.05478 (13)	0.84434 (5)	0.0159 (2)	
C5	0.27662 (14)	0.17456 (13)	0.83148 (5)	0.0163 (2)	
H5	0.254784	0.237297	0.867450	0.020*	
C6	0.23720 (14)	0.20155 (12)	0.76565 (5)	0.0157 (2)	
H6	0.186115	0.281924	0.756937	0.019*	
C7	0.23409 (15)	0.14359 (13)	0.64133 (5)	0.0176 (2)	
C8	0.35498 (18)	0.10742 (15)	0.96211 (5)	0.0244 (3)	
H8A	0.428596	0.227192	0.957834	0.037*	
H8B	0.389443	0.069884	1.004341	0.037*	
H8C	0.221074	0.085851	0.962013	0.037*	
C9	−0.29525 (14)	0.73995 (13)	−0.18604 (5)	0.0159 (2)	
C10	−0.19209 (15)	0.88110 (13)	−0.22204 (5)	0.0175 (2)	
H10	−0.088827	0.968468	−0.200711	0.021*	
C11	−0.23745 (15)	0.89642 (13)	−0.28853 (5)	0.0180 (2)	
H11	−0.165531	0.992937	−0.312675	0.022*	
C12	−0.38967 (15)	0.76852 (13)	−0.31932 (5)	0.0174 (2)	
C13	−0.49534 (15)	0.62658 (13)	−0.28356 (5)	0.0184 (2)	
H13	−0.599812	0.539892	−0.304622	0.022*	
C14	−0.44744 (15)	0.61277 (13)	−0.21759 (5)	0.0173 (2)	
H14	−0.518706	0.515903	−0.193521	0.021*	
C15	−0.23371 (15)	0.73027 (13)	−0.11581 (5)	0.0172 (2)	
C16	−0.34828 (18)	0.91561 (15)	−0.42219 (6)	0.0263 (3)	
H16A	−0.365021	1.011781	−0.401850	0.039*	
H16B	−0.397633	0.897616	−0.468488	0.039*	
H16C	−0.214467	0.936813	−0.422242	0.039*	
C17	0.26553 (17)	0.48170 (15)	0.50821 (6)	0.0252 (3)	
H17	0.347776	0.528136	0.545692	0.030*	
C18	0.29914 (17)	0.56891 (15)	0.44953 (6)	0.0238 (3)	
H18	0.403164	0.672865	0.446964	0.029*	
C19	0.18023 (15)	0.50428 (13)	0.39398 (5)	0.0168 (2)	
C20	0.03222 (17)	0.35114 (14)	0.40097 (6)	0.0226 (2)	
H20	−0.052170	0.301547	0.364326	0.027*	
C21	0.00822 (17)	0.27103 (14)	0.46161 (6)	0.0241 (3)	
H21	−0.093865	0.166285	0.465557	0.029*	
C22	−0.20434 (16)	0.45762 (14)	0.07493 (5)	0.0203 (2)	
H22	−0.314048	0.359357	0.066800	0.024*	
C23	−0.10326 (16)	0.47160 (13)	0.13413 (5)	0.0193 (2)	
H23	−0.143513	0.384225	0.165454	0.023*	
C24	0.05757 (15)	0.61441 (13)	0.14743 (5)	0.0165 (2)	
C25	0.11057 (15)	0.73538 (13)	0.09840 (5)	0.0189 (2)	
H25	0.220969	0.833944	0.104789	0.023*	
C26	0.00299 (16)	0.71201 (14)	0.04075 (5)	0.0198 (2)	
H26	0.042030	0.795985	0.007956	0.024*	
C27	0.21952 (16)	0.60443 (13)	0.33100 (5)	0.0203 (2)	
H27A	0.350222	0.627564	0.318157	0.024*	
H27B	0.213336	0.713050	0.342222	0.024*	
C28	0.09391 (15)	0.53060 (13)	0.26962 (5)	0.0171 (2)	
H28A	−0.035274	0.520828	0.278537	0.021*	
H28B	0.089535	0.418013	0.258752	0.021*	
C29	0.17452 (15)	0.64592 (13)	0.21094 (5)	0.0188 (2)	
H29A	0.202096	0.761447	0.226854	0.023*	
H29B	0.295721	0.640333	0.199359	0.023*	

### 4-Methoxybenzoic acid–1,3-bis(pyridin-4-yl)propane (2/1) (I) Atomic displacement parameters (Å^2^)

**Table:**

	*U*^11^	*U*^22^	*U*^33^	*U*^12^	*U*^13^	*U*^23^
O1	0.0278 (4)	0.0288 (4)	0.0157 (4)	0.0149 (3)	0.0010 (3)	0.0051 (3)
O2	0.0313 (4)	0.0287 (4)	0.0168 (4)	0.0133 (4)	0.0017 (3)	−0.0015 (3)
O3	0.0236 (4)	0.0241 (4)	0.0163 (4)	0.0128 (3)	−0.0020 (3)	0.0023 (3)
O4	0.0218 (4)	0.0214 (4)	0.0188 (4)	0.0059 (3)	−0.0023 (3)	0.0059 (3)
O5	0.0331 (5)	0.0214 (4)	0.0199 (4)	0.0034 (4)	−0.0091 (4)	0.0014 (3)
O6	0.0308 (4)	0.0205 (4)	0.0151 (4)	0.0068 (3)	−0.0054 (3)	0.0003 (3)
N1	0.0261 (5)	0.0240 (5)	0.0157 (4)	0.0126 (4)	−0.0012 (4)	0.0013 (4)
N2	0.0197 (4)	0.0230 (5)	0.0156 (4)	0.0100 (4)	−0.0013 (4)	0.0008 (4)
C1	0.0125 (4)	0.0152 (5)	0.0169 (5)	0.0021 (4)	−0.0002 (4)	0.0011 (4)
C2	0.0172 (5)	0.0170 (5)	0.0186 (5)	0.0053 (4)	0.0014 (4)	−0.0009 (4)
C3	0.0176 (5)	0.0170 (5)	0.0228 (5)	0.0087 (4)	0.0004 (4)	0.0023 (4)
C4	0.0129 (4)	0.0168 (5)	0.0158 (5)	0.0033 (4)	−0.0004 (4)	0.0027 (4)
C5	0.0162 (5)	0.0158 (5)	0.0161 (5)	0.0053 (4)	0.0010 (4)	0.0001 (4)
C6	0.0148 (5)	0.0137 (5)	0.0178 (5)	0.0046 (4)	0.0009 (4)	0.0027 (4)
C7	0.0161 (5)	0.0158 (5)	0.0171 (5)	0.0023 (4)	0.0012 (4)	0.0016 (4)
C8	0.0321 (6)	0.0304 (6)	0.0151 (5)	0.0169 (5)	−0.0011 (5)	0.0019 (4)
C9	0.0165 (5)	0.0168 (5)	0.0155 (5)	0.0077 (4)	0.0002 (4)	0.0004 (4)
C10	0.0166 (5)	0.0161 (5)	0.0182 (5)	0.0047 (4)	−0.0005 (4)	0.0003 (4)
C11	0.0182 (5)	0.0172 (5)	0.0175 (5)	0.0057 (4)	0.0018 (4)	0.0023 (4)
C12	0.0215 (5)	0.0197 (5)	0.0140 (5)	0.0113 (4)	−0.0013 (4)	−0.0010 (4)
C13	0.0184 (5)	0.0158 (5)	0.0192 (5)	0.0050 (4)	−0.0034 (4)	−0.0025 (4)
C14	0.0168 (5)	0.0154 (5)	0.0196 (5)	0.0062 (4)	0.0006 (4)	0.0015 (4)
C15	0.0182 (5)	0.0185 (5)	0.0166 (5)	0.0090 (4)	0.0004 (4)	0.0013 (4)
C16	0.0360 (7)	0.0253 (6)	0.0153 (5)	0.0095 (5)	−0.0002 (5)	0.0034 (4)
C17	0.0271 (6)	0.0292 (6)	0.0171 (5)	0.0092 (5)	−0.0063 (5)	−0.0007 (5)
C18	0.0238 (6)	0.0237 (6)	0.0190 (5)	0.0042 (5)	−0.0049 (5)	−0.0003 (4)
C19	0.0195 (5)	0.0188 (5)	0.0149 (5)	0.0108 (4)	−0.0011 (4)	−0.0003 (4)
C20	0.0242 (6)	0.0236 (6)	0.0166 (5)	0.0061 (5)	−0.0047 (4)	0.0002 (4)
C21	0.0265 (6)	0.0219 (5)	0.0198 (5)	0.0052 (5)	−0.0009 (5)	0.0025 (4)
C22	0.0190 (5)	0.0217 (5)	0.0192 (5)	0.0071 (4)	−0.0004 (4)	0.0013 (4)
C23	0.0203 (5)	0.0192 (5)	0.0179 (5)	0.0072 (4)	0.0016 (4)	0.0038 (4)
C24	0.0173 (5)	0.0206 (5)	0.0147 (5)	0.0109 (4)	0.0008 (4)	0.0015 (4)
C25	0.0175 (5)	0.0194 (5)	0.0190 (5)	0.0063 (4)	−0.0002 (4)	0.0026 (4)
C26	0.0209 (5)	0.0216 (5)	0.0175 (5)	0.0088 (4)	0.0017 (4)	0.0043 (4)
C27	0.0238 (5)	0.0185 (5)	0.0165 (5)	0.0061 (4)	−0.0032 (4)	0.0020 (4)
C28	0.0174 (5)	0.0182 (5)	0.0166 (5)	0.0078 (4)	−0.0015 (4)	0.0014 (4)
C29	0.0188 (5)	0.0195 (5)	0.0168 (5)	0.0062 (4)	−0.0027 (4)	0.0025 (4)

### 4-Methoxybenzoic acid–1,3-bis(pyridin-4-yl)propane (2/1) (I) Geometric parameters (Å, º)

**Table:**

O1—C7	1.3158 (15)	C11—C12	1.3914 (16)
O1—H1A	0.869 (17)	C11—H11	0.9500
O2—C7	1.2229 (13)	C12—C13	1.3995 (15)
O3—C4	1.3610 (14)	C13—C14	1.3814 (16)
O3—C8	1.4330 (13)	C13—H13	0.9500
O4—C15	1.3289 (13)	C14—H14	0.9500
O4—H4A	0.879 (16)	C16—H16A	0.9800
O5—C15	1.2132 (14)	C16—H16B	0.9800
O6—C12	1.3620 (14)	C16—H16C	0.9800
O6—C16	1.4356 (14)	C17—C18	1.3769 (17)
N1—C21	1.3330 (16)	C17—H17	0.9500
N1—C17	1.3382 (16)	C18—C19	1.3915 (16)
N1—H1B	0.889 (19)	C18—H18	0.9500
N2—C22	1.3360 (15)	C19—C20	1.3874 (16)
N2—C26	1.3470 (15)	C19—C27	1.5061 (15)
N2—H4B	0.87 (2)	C20—C21	1.3838 (17)
C1—C6	1.3928 (14)	C20—H20	0.9500
C1—C2	1.3938 (17)	C21—H21	0.9500
C1—C7	1.4864 (16)	C22—C23	1.3850 (17)
C2—C3	1.3786 (16)	C22—H22	0.9500
C2—H2	0.971 (13)	C23—C24	1.3910 (16)
C3—C4	1.4005 (15)	C23—H23	0.9500
C3—H3	0.9500	C24—C25	1.3936 (15)
C4—C5	1.3919 (16)	C24—C29	1.5043 (16)
C5—C6	1.3878 (15)	C25—C26	1.3755 (17)
C5—H5	0.9500	C25—H25	0.9500
C6—H6	0.9500	C26—H26	0.9500
C8—H8A	0.9800	C27—C28	1.5190 (16)
C8—H8B	0.9800	C27—H27A	0.9900
C8—H8C	0.9800	C27—H27B	0.9900
C9—C10	1.3930 (14)	C28—C29	1.5271 (15)
C9—C14	1.3947 (16)	C28—H28A	0.9900
C9—C15	1.4871 (16)	C28—H28B	0.9900
C10—C11	1.3882 (16)	C29—H29A	0.9900
C10—H10	0.9500	C29—H29B	0.9900
			
C7—O1—H1A	108.6 (17)	O5—C15—C9	122.12 (10)
C4—O3—C8	116.51 (9)	O4—C15—C9	114.09 (9)
C15—O4—H4A	111.9 (14)	O6—C16—H16A	109.5
C12—O6—C16	117.68 (9)	O6—C16—H16B	109.5
C21—N1—C17	117.75 (10)	H16A—C16—H16B	109.5
C21—N1—H1B	130 (3)	O6—C16—H16C	109.5
C17—N1—H1B	112 (3)	H16A—C16—H16C	109.5
C22—N2—C26	117.21 (10)	H16B—C16—H16C	109.5
C22—N2—H4B	130 (4)	N1—C17—C18	122.84 (11)
C26—N2—H4B	113 (4)	N1—C17—H17	118.6
C6—C1—C2	118.66 (10)	C18—C17—H17	118.6
C6—C1—C7	121.65 (10)	C17—C18—C19	119.78 (11)
C2—C1—C7	119.69 (9)	C17—C18—H18	120.1
C3—C2—C1	121.08 (10)	C19—C18—H18	120.1
C3—C2—H2	119.2 (9)	C20—C19—C18	117.10 (10)
C1—C2—H2	119.7 (9)	C20—C19—C27	124.59 (10)
C2—C3—C4	119.70 (11)	C18—C19—C27	118.31 (10)
C2—C3—H3	120.1	C21—C20—C19	119.63 (11)
C4—C3—H3	120.1	C21—C20—H20	120.2
O3—C4—C5	124.33 (9)	C19—C20—H20	120.2
O3—C4—C3	115.68 (10)	N1—C21—C20	122.90 (11)
C5—C4—C3	119.98 (10)	N1—C21—H21	118.6
C6—C5—C4	119.44 (10)	C20—C21—H21	118.6
C6—C5—H5	120.3	N2—C22—C23	123.27 (11)
C4—C5—H5	120.3	N2—C22—H22	118.4
C5—C6—C1	121.13 (10)	C23—C22—H22	118.4
C5—C6—H6	119.4	C22—C23—C24	119.58 (11)
C1—C6—H6	119.4	C22—C23—H23	120.2
O2—C7—O1	123.73 (11)	C24—C23—H23	120.2
O2—C7—C1	122.00 (11)	C23—C24—C25	116.96 (11)
O1—C7—C1	114.28 (9)	C23—C24—C29	124.26 (10)
O3—C8—H8A	109.5	C25—C24—C29	118.78 (10)
O3—C8—H8B	109.5	C26—C25—C24	119.98 (10)
H8A—C8—H8B	109.5	C26—C25—H25	120.0
O3—C8—H8C	109.5	C24—C25—H25	120.0
H8A—C8—H8C	109.5	N2—C26—C25	122.97 (10)
H8B—C8—H8C	109.5	N2—C26—H26	118.5
C10—C9—C14	119.04 (10)	C25—C26—H26	118.5
C10—C9—C15	117.96 (10)	C19—C27—C28	118.18 (9)
C14—C9—C15	122.98 (10)	C19—C27—H27A	107.8
C11—C10—C9	121.19 (10)	C28—C27—H27A	107.8
C11—C10—H10	119.4	C19—C27—H27B	107.8
C9—C10—H10	119.4	C28—C27—H27B	107.8
C10—C11—C12	119.11 (10)	H27A—C27—H27B	107.1
C10—C11—H11	120.4	C27—C28—C29	108.07 (9)
C12—C11—H11	120.4	C27—C28—H28A	110.1
O6—C12—C11	124.28 (10)	C29—C28—H28A	110.1
O6—C12—C13	115.45 (10)	C27—C28—H28B	110.1
C11—C12—C13	120.27 (10)	C29—C28—H28B	110.1
C14—C13—C12	119.88 (10)	H28A—C28—H28B	108.4
C14—C13—H13	120.1	C24—C29—C28	117.92 (9)
C12—C13—H13	120.1	C24—C29—H29A	107.8
C13—C14—C9	120.51 (10)	C28—C29—H29A	107.8
C13—C14—H14	119.7	C24—C29—H29B	107.8
C9—C14—H14	119.7	C28—C29—H29B	107.8
O5—C15—O4	123.78 (11)	H29A—C29—H29B	107.2
			
C6—C1—C2—C3	0.30 (16)	C15—C9—C14—C13	−178.28 (10)
C7—C1—C2—C3	179.49 (9)	C10—C9—C15—O5	7.14 (16)
C1—C2—C3—C4	−0.95 (16)	C14—C9—C15—O5	−174.62 (10)
C8—O3—C4—C5	2.42 (14)	C10—C9—C15—O4	−172.09 (9)
C8—O3—C4—C3	−178.29 (9)	C14—C9—C15—O4	6.15 (15)
C2—C3—C4—O3	−178.75 (9)	C21—N1—C17—C18	0.08 (18)
C2—C3—C4—C5	0.57 (15)	N1—C17—C18—C19	0.39 (19)
O3—C4—C5—C6	179.70 (9)	C17—C18—C19—C20	−0.65 (17)
C3—C4—C5—C6	0.44 (15)	C17—C18—C19—C27	179.03 (11)
C4—C5—C6—C1	−1.10 (15)	C18—C19—C20—C21	0.48 (17)
C2—C1—C6—C5	0.73 (15)	C27—C19—C20—C21	−179.17 (11)
C7—C1—C6—C5	−178.44 (9)	C17—N1—C21—C20	−0.26 (18)
C6—C1—C7—O2	166.69 (10)	C19—C20—C21—N1	−0.03 (19)
C2—C1—C7—O2	−12.48 (15)	C26—N2—C22—C23	1.34 (16)
C6—C1—C7—O1	−13.18 (14)	N2—C22—C23—C24	0.23 (17)
C2—C1—C7—O1	167.66 (9)	C22—C23—C24—C25	−1.59 (15)
C14—C9—C10—C11	−0.44 (16)	C22—C23—C24—C29	177.80 (10)
C15—C9—C10—C11	177.87 (10)	C23—C24—C25—C26	1.39 (15)
C9—C10—C11—C12	0.44 (16)	C29—C24—C25—C26	−178.03 (10)
C16—O6—C12—C11	−2.11 (16)	C22—N2—C26—C25	−1.55 (16)
C16—O6—C12—C13	177.66 (9)	C24—C25—C26—N2	0.18 (17)
C10—C11—C12—O6	179.81 (10)	C20—C19—C27—C28	−2.62 (17)
C10—C11—C12—C13	0.04 (16)	C18—C19—C27—C28	177.73 (10)
O6—C12—C13—C14	179.69 (10)	C19—C27—C28—C29	−173.98 (9)
C11—C12—C13—C14	−0.52 (16)	C23—C24—C29—C28	−11.68 (16)
C12—C13—C14—C9	0.53 (16)	C25—C24—C29—C28	167.70 (9)
C10—C9—C14—C13	−0.05 (16)	C27—C28—C29—C24	−170.16 (9)

### 4-Methoxybenzoic acid–1,3-bis(pyridin-4-yl)propane (2/1) (I) Hydrogen-bond geometry (Å, º)

*Cg*2, *Cg*3 and *Cg*4 are the centroids of the N2/C22–C26, C1–C6 and C9–C14 rings, respectively.

**Table:**

*D*—H···*A*	*D*—H	H···*A*	*D*···*A*	*D*—H···*A*
O1—H1*A*···N1	0.87 (2)	1.71 (2)	2.5730 (16)	170 (3)
O4—H4*A*···N2	0.88 (2)	1.80 (2)	2.6721 (17)	171 (2)
N1—H1*B*···O1	0.89 (2)	1.69 (4)	2.5730 (16)	171 (5)
N2—H4*B*···O4	0.87 (2)	1.80 (4)	2.6720 (16)	177 (7)
C17—H17···O6^i^	0.95	2.48	3.342 (2)	151
C18—H18···O2^ii^	0.95	2.60	3.515 (2)	162
C21—H21···O2^iii^	0.95	2.43	3.2563 (19)	145
C26—H26···O5	0.95	2.36	3.0890 (18)	133
C3—H3···*Cg*4^iv^	0.95	2.64	3.4265 (19)	140
C5—H5···*Cg*2^v^	0.95	2.71	3.5440 (18)	146
C10—H10···*Cg*3^vi^	0.95	2.89	3.5859 (19)	131
C28—H28*B*···*Cg*4^vii^	0.99	2.91	3.7154 (19)	139

Symmetry codes: (i) *x*+1, *y*, *z*+1; (ii) −*x*+1, −*y*+1, −*z*+1; (iii) −*x*, −*y*, −*z*+1; (iv) *x*+1, *y*−1, *z*+1; (v) −*x*, −*y*+1, −*z*+1; (vi) *x*, *y*+1, *z*−1; (vii) −*x*, −*y*+1, −*z*.

### Biphenyl-4,4'-dicarboxylic acid–4-methoxypyridine (1/2) (II) Crystal data

**Table:**

C_14_H_9.43_O_4_·C_6_H_7.32_NO·C_6_H_7.25_NO	*F*(000) = 968.00
*M**_r_* = 460.47	*D*_x_ = 1.395 Mg m^−^^3^
Monoclinic, *P*2_1_/*c*	Mo *K*α radiation, λ = 0.71075 Å
*a* = 18.354 (6) Å	Cell parameters from 20884 reflections
*b* = 7.4166 (16) Å	θ = 3.0–33.0°
*c* = 16.674 (5) Å	µ = 0.10 mm^−^^1^
β = 104.943 (12)°	*T* = 93 K
*V* = 2192.9 (10) Å^3^	Block, colorless
*Z* = 4	0.45 × 0.40 × 0.35 mm

### Biphenyl-4,4'-dicarboxylic acid–4-methoxypyridine (1/2) (II) Data collection

**Table:**

Rigaku R-AXIS RAPID II diffractometer	4085 reflections with *I* > 2σ(*I*)
Detector resolution: 10.000 pixels mm^-1^	*R*_int_ = 0.035
ω scans	θ_max_ = 27.5°, θ_min_ = 3.0°
Absorption correction: numerical (NUMABS; Higashi, 1999)	*h* = −23→23
*T*_min_ = 0.963, *T*_max_ = 0.966	*k* = −8→9
20851 measured reflections	*l* = −21→21
5026 independent reflections	

### Biphenyl-4,4'-dicarboxylic acid–4-methoxypyridine (1/2) (II) Refinement

**Table:**

Refinement on *F*^2^	Primary atom site location: structure-invariant direct methods
Least-squares matrix: full	Secondary atom site location: difference Fourier map
*R*[*F*^2^ > 2σ(*F*^2^)] = 0.038	Hydrogen site location: mixed
*wR*(*F*^2^) = 0.108	H atoms treated by a mixture of independent and constrained refinement
*S* = 1.06	*w* = 1/[σ^2^(*F*_o_^2^) + (0.0656*P*)^2^ + 0.1824*P*] where *P* = (*F*_o_^2^ + 2*F*_c_^2^)/3
5026 reflections	(Δ/σ)_max_ = 0.001
323 parameters	Δρ_max_ = 0.35 e Å^−^^3^
4 restraints	Δρ_min_ = −0.26 e Å^−^^3^

### Biphenyl-4,4'-dicarboxylic acid–4-methoxypyridine (1/2) (II) Special details

**Table:**

Geometry. All esds (except the esd in the dihedral angle between two l.s. planes) are estimated using the full covariance matrix. The cell esds are taken into account individually in the estimation of esds in distances, angles and torsion angles; correlations between esds in cell parameters are only used when they are defined by crystal symmetry. An approximate (isotropic) treatment of cell esds is used for estimating esds involving l.s. planes.
Refinement. Reflections were merged by SHELXL according to the crystal class for the calculation of statistics and refinement. _reflns_Friedel_fraction is defined as the number of unique Friedel pairs measured divided by the number that would be possible theoretically, ignoring centric projections and systematic absences.

### Biphenyl-4,4'-dicarboxylic acid–4-methoxypyridine (1/2) (II) Fractional atomic coordinates and isotropic or equivalent isotropic displacement parameters (Å^2^)

**Table:**

	*x*	*y*	*z*	*U*_iso_*/*U*_eq_	Occ. (<1)
O1	0.50808 (4)	0.28798 (11)	0.54421 (5)	0.02581 (19)	
H1A	0.4857 (13)	0.300 (3)	0.5837 (12)	0.039*	0.68 (3)
O2	0.59317 (5)	0.45187 (11)	0.63520 (5)	0.02892 (19)	
O3	0.97286 (4)	0.40830 (10)	0.19753 (5)	0.02486 (18)	
H3A	0.9981 (11)	0.387 (3)	0.1612 (11)	0.037*	0.75 (3)
O4	0.89523 (5)	0.21022 (11)	0.11695 (5)	0.02858 (19)	
O5	0.31541 (4)	0.32860 (10)	0.83543 (5)	0.02399 (18)	
O6	1.16611 (4)	0.33625 (10)	−0.09371 (5)	0.02332 (18)	
N1	0.43554 (5)	0.29753 (12)	0.65825 (6)	0.0224 (2)	
H1B	0.457 (2)	0.279 (6)	0.618 (2)	0.034*	0.32 (3)
N2	1.05080 (5)	0.36571 (12)	0.08839 (6)	0.0214 (2)	
H3B	1.029 (3)	0.375 (8)	0.130 (3)	0.032*	0.25 (3)
C1	0.61880 (6)	0.37495 (13)	0.50687 (6)	0.0189 (2)	
C2	0.68583 (6)	0.47177 (14)	0.52414 (6)	0.0209 (2)	
H2	0.6997	0.5436	0.5729	0.025*	
C3	0.73242 (6)	0.46464 (14)	0.47108 (6)	0.0217 (2)	
H3	0.7783	0.5308	0.4844	0.026*	
C4	0.71359 (6)	0.36215 (13)	0.39803 (6)	0.0174 (2)	
C5	0.64529 (6)	0.26812 (15)	0.38096 (7)	0.0234 (2)	
H5	0.6305	0.1986	0.3316	0.028*	
C6	0.59875 (6)	0.27387 (15)	0.43431 (7)	0.0244 (2)	
H6	0.5527	0.2082	0.4212	0.029*	
C7	0.57184 (6)	0.37705 (13)	0.56804 (6)	0.0200 (2)	
C8	0.86113 (6)	0.32919 (13)	0.23465 (6)	0.0194 (2)	
C9	0.79105 (6)	0.24541 (14)	0.21431 (6)	0.0214 (2)	
H9	0.7757	0.1788	0.1641	0.026*	
C10	0.74320 (6)	0.25758 (14)	0.26609 (6)	0.0206 (2)	
H10	0.6954	0.2000	0.2505	0.025*	
C11	0.76402 (6)	0.35335 (13)	0.34110 (6)	0.0178 (2)	
C12	0.83442 (6)	0.43886 (14)	0.36029 (7)	0.0224 (2)	
H12	0.8499	0.5057	0.4104	0.027*	
C13	0.88208 (6)	0.42841 (14)	0.30799 (7)	0.0222 (2)	
H13	0.9292	0.4892	0.3222	0.027*	
C14	0.91111 (6)	0.30991 (13)	0.17715 (7)	0.0204 (2)	
C15	0.37013 (6)	0.21013 (14)	0.65102 (7)	0.0233 (2)	
H15	0.3508	0.1389	0.6029	0.028*	
C16	0.32982 (6)	0.21856 (14)	0.70967 (7)	0.0232 (2)	
H16	0.2839	0.1538	0.7023	0.028*	
C17	0.35741 (6)	0.32408 (13)	0.78043 (6)	0.0198 (2)	
C18	0.42461 (6)	0.41775 (14)	0.78814 (6)	0.0222 (2)	
H18	0.4448	0.4921	0.8350	0.027*	
C19	0.46117 (6)	0.39954 (15)	0.72562 (7)	0.0240 (2)	
H19	0.5071	0.4632	0.7309	0.029*	
C20	0.34345 (7)	0.43321 (17)	0.90933 (7)	0.0282 (2)	
H20A	0.3074	0.4280	0.9436	0.042*	
H20B	0.3500	0.5587	0.8942	0.042*	
H20C	0.3921	0.3843	0.9406	0.042*	
C21	1.11352 (6)	0.46206 (14)	0.09146 (7)	0.0240 (2)	
H21	1.1321	0.5391	0.1377	0.029*	
C22	1.15210 (6)	0.45467 (14)	0.03100 (7)	0.0236 (2)	
H22	1.1959	0.5262	0.0353	0.028*	
C23	1.12592 (6)	0.34022 (13)	−0.03684 (6)	0.0192 (2)	
C24	1.06118 (6)	0.23911 (14)	−0.04050 (7)	0.0210 (2)	
H24	1.0418	0.1591	−0.0855	0.025*	
C25	1.02579 (6)	0.25802 (14)	0.02294 (7)	0.0221 (2)	
H25	0.9811	0.1905	0.0197	0.027*	
C26	1.13950 (7)	0.21987 (15)	−0.16463 (7)	0.0261 (2)	
H26A	1.0887	0.2570	−0.1952	0.039*	
H26B	1.1382	0.0950	−0.1458	0.039*	
H26C	1.1736	0.2285	−0.2011	0.039*	

### Biphenyl-4,4'-dicarboxylic acid–4-methoxypyridine (1/2) (II) Atomic displacement parameters (Å^2^)

**Table:**

	*U*^11^	*U*^22^	*U*^33^	*U*^12^	*U*^13^	*U*^23^
O1	0.0224 (4)	0.0336 (4)	0.0239 (4)	−0.0043 (3)	0.0104 (3)	−0.0059 (3)
O2	0.0271 (4)	0.0399 (5)	0.0216 (4)	−0.0053 (3)	0.0095 (3)	−0.0067 (3)
O3	0.0229 (4)	0.0294 (4)	0.0249 (4)	−0.0019 (3)	0.0108 (3)	−0.0029 (3)
O4	0.0351 (5)	0.0281 (4)	0.0277 (4)	−0.0069 (3)	0.0176 (4)	−0.0069 (3)
O5	0.0257 (4)	0.0266 (4)	0.0228 (4)	−0.0030 (3)	0.0119 (3)	−0.0018 (3)
O6	0.0227 (4)	0.0254 (4)	0.0245 (4)	−0.0023 (3)	0.0109 (3)	−0.0028 (3)
N1	0.0239 (5)	0.0245 (4)	0.0211 (4)	0.0038 (4)	0.0096 (4)	0.0032 (4)
N2	0.0214 (4)	0.0209 (4)	0.0237 (5)	0.0025 (3)	0.0092 (4)	0.0015 (3)
C1	0.0203 (5)	0.0185 (5)	0.0182 (5)	0.0041 (4)	0.0057 (4)	0.0032 (4)
C2	0.0238 (5)	0.0215 (5)	0.0170 (4)	0.0001 (4)	0.0047 (4)	−0.0017 (4)
C3	0.0202 (5)	0.0240 (5)	0.0207 (5)	−0.0034 (4)	0.0051 (4)	−0.0012 (4)
C4	0.0187 (5)	0.0161 (4)	0.0172 (5)	0.0037 (4)	0.0044 (4)	0.0027 (4)
C5	0.0243 (5)	0.0260 (5)	0.0209 (5)	−0.0037 (4)	0.0076 (4)	−0.0057 (4)
C6	0.0212 (5)	0.0290 (5)	0.0245 (5)	−0.0053 (4)	0.0083 (4)	−0.0051 (4)
C7	0.0198 (5)	0.0209 (5)	0.0196 (5)	0.0037 (4)	0.0055 (4)	0.0025 (4)
C8	0.0222 (5)	0.0178 (4)	0.0194 (5)	0.0029 (4)	0.0075 (4)	0.0030 (4)
C9	0.0238 (5)	0.0227 (5)	0.0172 (5)	0.0002 (4)	0.0047 (4)	−0.0021 (4)
C10	0.0186 (5)	0.0229 (5)	0.0201 (5)	−0.0010 (4)	0.0047 (4)	−0.0004 (4)
C11	0.0191 (5)	0.0160 (4)	0.0183 (5)	0.0033 (4)	0.0051 (4)	0.0024 (4)
C12	0.0239 (5)	0.0231 (5)	0.0209 (5)	−0.0020 (4)	0.0069 (4)	−0.0043 (4)
C13	0.0205 (5)	0.0234 (5)	0.0233 (5)	−0.0026 (4)	0.0072 (4)	−0.0014 (4)
C14	0.0232 (5)	0.0182 (5)	0.0210 (5)	0.0020 (4)	0.0081 (4)	0.0031 (4)
C15	0.0288 (6)	0.0205 (5)	0.0209 (5)	0.0009 (4)	0.0071 (4)	−0.0004 (4)
C16	0.0246 (5)	0.0206 (5)	0.0255 (5)	−0.0026 (4)	0.0087 (4)	0.0003 (4)
C17	0.0215 (5)	0.0191 (5)	0.0203 (5)	0.0037 (4)	0.0082 (4)	0.0048 (4)
C18	0.0211 (5)	0.0254 (5)	0.0199 (5)	0.0004 (4)	0.0051 (4)	0.0008 (4)
C19	0.0213 (5)	0.0279 (5)	0.0234 (5)	0.0007 (4)	0.0070 (4)	0.0022 (4)
C20	0.0280 (6)	0.0383 (6)	0.0206 (5)	−0.0024 (5)	0.0103 (4)	−0.0039 (5)
C21	0.0244 (5)	0.0237 (5)	0.0249 (5)	−0.0014 (4)	0.0077 (4)	−0.0042 (4)
C22	0.0204 (5)	0.0230 (5)	0.0280 (5)	−0.0031 (4)	0.0075 (4)	−0.0021 (4)
C23	0.0184 (5)	0.0181 (5)	0.0222 (5)	0.0042 (4)	0.0070 (4)	0.0038 (4)
C24	0.0204 (5)	0.0201 (5)	0.0226 (5)	−0.0006 (4)	0.0057 (4)	−0.0003 (4)
C25	0.0198 (5)	0.0200 (5)	0.0270 (5)	−0.0002 (4)	0.0069 (4)	0.0032 (4)
C26	0.0307 (6)	0.0246 (5)	0.0255 (5)	−0.0005 (4)	0.0114 (5)	−0.0036 (4)

### Biphenyl-4,4'-dicarboxylic acid–4-methoxypyridine (1/2) (II) Geometric parameters (Å, º)

**Table:**

O1—C7	1.3130 (13)	C9—C10	1.3841 (15)
O1—H1A	0.866 (17)	C9—H9	0.9500
O2—C7	1.2202 (13)	C10—C11	1.4029 (14)
O3—C14	1.3168 (13)	C10—H10	0.9500
O3—H3A	0.868 (16)	C11—C12	1.4003 (15)
O4—C14	1.2198 (13)	C12—C13	1.3875 (15)
O5—C17	1.3420 (13)	C12—H12	0.9500
O5—C20	1.4345 (13)	C13—H13	0.9500
O6—C23	1.3435 (13)	C15—C16	1.3715 (16)
O6—C26	1.4436 (13)	C15—H15	0.9500
N1—C19	1.3353 (15)	C16—C17	1.3974 (15)
N1—C15	1.3422 (15)	C16—H16	0.9500
N1—H1B	0.88 (2)	C17—C18	1.3924 (15)
N2—C25	1.3347 (14)	C18—C19	1.3841 (16)
N2—C21	1.3446 (15)	C18—H18	0.9500
N2—H3B	0.88 (2)	C19—H19	0.9500
C1—C2	1.3890 (15)	C20—H20A	0.9800
C1—C6	1.3899 (15)	C20—H20B	0.9800
C1—C7	1.4955 (15)	C20—H20C	0.9800
C2—C3	1.3809 (15)	C21—C22	1.3743 (16)
C2—H2	0.9500	C21—H21	0.9500
C3—C4	1.4013 (14)	C22—C23	1.3967 (15)
C3—H3	0.9500	C22—H22	0.9500
C4—C5	1.3981 (15)	C23—C24	1.3931 (15)
C4—C11	1.4885 (15)	C24—C25	1.3838 (16)
C5—C6	1.3837 (15)	C24—H24	0.9500
C5—H5	0.9500	C25—H25	0.9500
C6—H6	0.9500	C26—H26A	0.9800
C8—C9	1.3894 (15)	C26—H26B	0.9800
C8—C13	1.3937 (15)	C26—H26C	0.9800
C8—C14	1.4949 (15)		
			
C7—O1—H1A	106.0 (15)	C12—C13—H13	119.8
C14—O3—H3A	107.3 (14)	C8—C13—H13	119.8
C17—O5—C20	117.29 (9)	O4—C14—O3	123.73 (10)
C23—O6—C26	117.38 (8)	O4—C14—C8	121.99 (10)
C19—N1—C15	117.52 (10)	O3—C14—C8	114.27 (9)
C19—N1—H1B	127 (3)	N1—C15—C16	123.26 (10)
C15—N1—H1B	115 (3)	N1—C15—H15	118.4
C25—N2—C21	117.36 (10)	C16—C15—H15	118.4
C25—N2—H3B	124 (4)	C15—C16—C17	118.88 (10)
C21—N2—H3B	119 (4)	C15—C16—H16	120.6
C2—C1—C6	118.75 (10)	C17—C16—H16	120.6
C2—C1—C7	119.25 (9)	O5—C17—C18	125.15 (10)
C6—C1—C7	121.97 (9)	O5—C17—C16	116.36 (9)
C3—C2—C1	120.60 (9)	C18—C17—C16	118.48 (10)
C3—C2—H2	119.7	C19—C18—C17	118.15 (10)
C1—C2—H2	119.7	C19—C18—H18	120.9
C2—C3—C4	121.53 (10)	C17—C18—H18	120.9
C2—C3—H3	119.2	N1—C19—C18	123.71 (10)
C4—C3—H3	119.2	N1—C19—H19	118.1
C5—C4—C3	117.05 (9)	C18—C19—H19	118.1
C5—C4—C11	121.38 (9)	O5—C20—H20A	109.5
C3—C4—C11	121.57 (9)	O5—C20—H20B	109.5
C6—C5—C4	121.57 (10)	H20A—C20—H20B	109.5
C6—C5—H5	119.2	O5—C20—H20C	109.5
C4—C5—H5	119.2	H20A—C20—H20C	109.5
C5—C6—C1	120.49 (10)	H20B—C20—H20C	109.5
C5—C6—H6	119.8	N2—C21—C22	123.29 (10)
C1—C6—H6	119.8	N2—C21—H21	118.4
O2—C7—O1	123.81 (10)	C22—C21—H21	118.4
O2—C7—C1	121.71 (10)	C21—C22—C23	118.92 (10)
O1—C7—C1	114.45 (9)	C21—C22—H22	120.5
C9—C8—C13	118.61 (10)	C23—C22—H22	120.5
C9—C8—C14	118.90 (9)	O6—C23—C24	125.08 (9)
C13—C8—C14	122.50 (9)	O6—C23—C22	116.62 (9)
C10—C9—C8	121.00 (10)	C24—C23—C22	118.30 (10)
C10—C9—H9	119.5	C25—C24—C23	118.37 (10)
C8—C9—H9	119.5	C25—C24—H24	120.8
C9—C10—C11	121.21 (10)	C23—C24—H24	120.8
C9—C10—H10	119.4	N2—C25—C24	123.75 (10)
C11—C10—H10	119.4	N2—C25—H25	118.1
C12—C11—C10	117.15 (10)	C24—C25—H25	118.1
C12—C11—C4	121.55 (9)	O6—C26—H26A	109.5
C10—C11—C4	121.30 (9)	O6—C26—H26B	109.5
C13—C12—C11	121.68 (10)	H26A—C26—H26B	109.5
C13—C12—H12	119.2	O6—C26—H26C	109.5
C11—C12—H12	119.2	H26A—C26—H26C	109.5
C12—C13—C8	120.33 (10)	H26B—C26—H26C	109.5
			
C6—C1—C2—C3	−1.35 (15)	C9—C8—C13—C12	1.60 (15)
C7—C1—C2—C3	176.47 (9)	C14—C8—C13—C12	−178.50 (9)
C1—C2—C3—C4	0.75 (15)	C9—C8—C14—O4	−6.65 (15)
C2—C3—C4—C5	0.35 (15)	C13—C8—C14—O4	173.45 (10)
C2—C3—C4—C11	−179.56 (9)	C9—C8—C14—O3	173.64 (9)
C3—C4—C5—C6	−0.85 (15)	C13—C8—C14—O3	−6.26 (14)
C11—C4—C5—C6	179.06 (10)	C19—N1—C15—C16	−1.16 (15)
C4—C5—C6—C1	0.25 (17)	N1—C15—C16—C17	0.51 (16)
C2—C1—C6—C5	0.86 (16)	C20—O5—C17—C18	−2.33 (14)
C7—C1—C6—C5	−176.90 (9)	C20—O5—C17—C16	178.78 (9)
C2—C1—C7—O2	−4.87 (15)	C15—C16—C17—O5	179.48 (9)
C6—C1—C7—O2	172.88 (10)	C15—C16—C17—C18	0.51 (15)
C2—C1—C7—O1	177.12 (9)	O5—C17—C18—C19	−179.69 (9)
C6—C1—C7—O1	−5.13 (14)	C16—C17—C18—C19	−0.81 (15)
C13—C8—C9—C10	−0.89 (15)	C15—N1—C19—C18	0.82 (15)
C14—C8—C9—C10	179.21 (9)	C17—C18—C19—N1	0.15 (16)
C8—C9—C10—C11	−0.58 (15)	C25—N2—C21—C22	0.17 (15)
C9—C10—C11—C12	1.30 (15)	N2—C21—C22—C23	−0.80 (16)
C9—C10—C11—C4	−178.20 (9)	C26—O6—C23—C24	0.86 (14)
C5—C4—C11—C12	−176.28 (10)	C26—O6—C23—C22	−179.47 (9)
C3—C4—C11—C12	3.63 (14)	C21—C22—C23—O6	−179.22 (9)
C5—C4—C11—C10	3.20 (14)	C21—C22—C23—C24	0.47 (15)
C3—C4—C11—C10	−176.89 (9)	O6—C23—C24—C25	−179.92 (9)
C10—C11—C12—C13	−0.58 (15)	C22—C23—C24—C25	0.42 (14)
C4—C11—C12—C13	178.92 (9)	C21—N2—C25—C24	0.82 (15)
C11—C12—C13—C8	−0.87 (16)	C23—C24—C25—N2	−1.12 (15)

### Biphenyl-4,4'-dicarboxylic acid–4-methoxypyridine (1/2) (II) Hydrogen-bond geometry (Å, º)

*Cg*2 and *Cg*4 are the centroids of the C8–C13 and N2/C21–C25 rings, respectively.

**Table:**

*D*—H···*A*	*D*—H	H···*A*	*D*···*A*	*D*—H···*A*
O1—H1*A*···N1	0.87 (2)	1.73 (2)	2.5882 (15)	173 (2)
O3—H3*A*···N2	0.87 (2)	1.74 (2)	2.6078 (15)	175 (2)
N1—H1*B*···O1	0.88 (2)	1.73 (5)	2.5882 (16)	167 (5)
N2—H3*B*···O3	0.88 (2)	1.74 (6)	2.6077 (15)	169 (5)
C10—H10···O2^i^	0.95	2.57	3.4146 (17)	148
C19—H19···O2	0.95	2.52	3.1901 (17)	128
C20—H20*A*···O6^ii^	0.98	2.60	3.3210 (18)	131
C25—H25···O4	0.95	2.54	3.2035 (17)	127
C26—H26*B*···O4^iii^	0.98	2.43	3.3874 (17)	167
C12—H12···*Cg*4^iv^	0.95	2.90	3.6968 (16)	142
C21—H21···*Cg*2^iv^	0.95	2.64	3.5284 (16)	155

Symmetry codes: (i) *x*, −*y*+1/2, *z*−1/2; (ii) *x*−1, *y*, *z*+1; (iii) −*x*+2, −*y*, −*z*; (iv) −*x*+2, *y*+1/2, −*z*+1/2.
